# Translational Validation of a Novel Multi-Locus ctDNA Methylation Assay for Early Detection and Stratification of Colorectal Cancer: An Exploratory Prospective, Case-Control Study

**DOI:** 10.3390/ijms27135738

**Published:** 2026-06-25

**Authors:** Hayoung Lee, Jae Cheol Kang, In Ja Park, Gwang-un Kim, Hwi Hyun, Na Young Min, Sungwon Jeon, Byoung-Chul Kim

**Affiliations:** 1 Department of Colon and Rectal Surgery, Asan Medical Center, University of Ulsan College of Medicine, Seoul 05505, Republic of Korea; cbya1322@naver.com (H.L.); jaecheols92@gmail.com (J.C.K.); 2 Department of Surgery, Kyung Hee University Hospital at Gangdong, Kyung Hee University School of Medicine, Seoul 02447, Republic of Korea; 3 Health Screening and Promotion Center, Asan Medical Center, University of Ulsan College of Medicine, Seoul 05505, Republic of Korea; mvlight@amc.seoul.kr; 4 Clinomics Inc., Ulsan 44919, Republic of Korea; h1208h@clinomics.co.kr (H.H.); ny.min@evergenome.com (N.Y.M.); jsungwon@clinomics.co.kr (S.J.); bcghim@clinomics.co.kr (B.-C.K.); 5 Evergenome Inc., Eungubinam-ro 33beon-gil, Jijeok-dong, Yuseong-gu, Daejeon 34086, Republic of Korea; 6 AgingLab Inc., Ulsan 44919, Republic of Korea

**Keywords:** colorectal neoplasms, liquid biopsy, DNA methylation

## Abstract

To evaluate the diagnostic performance and clinicopathologic relevance of a multi-locus circulating tumor DNA methylation assay, in this prospective, single-center, case-control exploratory study, we enrolled 35 patients with colorectal cancer undergoing surgery and 57 healthy controls undergoing screening colonoscopy at the Asan Medical Center, Seoul, Republic of Korea between July 2024 and January 2025. Peripheral blood was collected before surgery or colonoscopy, and circulating tumor DNA methylation was analyzed using a multi-locus panel targeting Septin9, IKZF1, BCAT1, Septin9-2, BCAN, and VAV3. The main outcomes were test accuracy (sensitivity, specificity, and area under the curve [AUC]) and associations between methylation marker positivity and clinicopathologic features. Circulating tumor DNA was positive in 74.3% of the patients and 12.3% of controls, yielding a sensitivity of 74.3%, specificity of 87.7%, and an AUC of 0.837, whereas serum carcinoembryonic antigen exhibited lower sensitivity (25.7%). Sensitivity in stage I disease was limited (36.4%). Circulating tumor DNA-positive tumors were larger (5.7 cm vs. 2.2 cm, *p* < 0.001) and had more advanced T and N stages. The number of positive markers increased with pathologic stage (*p* = 0.003). Individual marker analysis revealed that BCAT1, Septin9-2, and VAV3 were associated with higher T stage, whereas BCAN positivity was linked to nodal metastasis. The six-marker circulating tumor DNA methylation assay demonstrated acceptable diagnostic accuracy, with multi-locus patterns associated with tumor burden and invasive features. However, sensitivity for early-stage disease was limited. The assay may serve as a complementary tool for screening and risk stratification.

## 1. Introduction

Colorectal cancer (CRC) remains a major global health burden, ranking as the third most commonly diagnosed malignancy and the second leading cause of cancer-related deaths worldwide. In South Korea, it is also among the most prevalent cancers and a leading cause of cancer mortality [1,2]. In Korea, a nationwide CRC screening program was first implemented in 2004, initially centered on fecal occult blood testing (FOBT) with colonoscopy used for subsequent diagnostic confirmation. Over time, colonoscopy has been pilot-tested and increasingly emphasized as a primary screening modality [3]. Overall, this national screening program has contributed to a gradual decline in age-specific mortality over the past decade [4,5].

Despite these achievements, current screening tools have several limitations that present an unmet clinical need for a non-invasive, high-performing alternative. Colonoscopy is invasive, resource-intensive, and subject to inter-operator variability in adenoma detection rates [6]. Stool-based tests, including the fecal immunochemical test (FIT) and FOBT, while noninvasive, suffer from low sensitivity for early-stage disease and limited patient participation, particularly in routine surveillance [7,8]. Furthermore, conventional blood-based biomarkers like serum carcinoembryonic antigen (CEA) demonstrate poor sensitivity and specificity for primary detection [9,10]. In contrast, DNA methylation-based assays targeting circulating tumor DNA (ctDNA) have emerged as a promising diagnostic approach. Aberrant DNA methylation is one of the earliest molecular events in colorectal carcinogenesis, offering a biologically plausible and potentially more sensitive means of early detection [11].

While previous single-marker ctDNA methylation assays, such as those targeting Septin9 alone, have demonstrated moderate performance and even received regulatory approval, malignancies typically exhibit considerable epigenetic heterogeneity [12,13,14,15,16]. This heterogeneity means that no single methylation locus is universally altered across all CRC subtypes and stages; consequently, single-marker approaches frequently fail to capture the full spectrum of methylation alterations, resulting in insufficient sensitivity, particularly for early-stage disease.

To address this critical technical limitation, multi-locus methylation panels have been proposed across various cancer types, aiming to substantially improve sensitivity while preserving specificity [17,18,19]. Our work represents a crucial translational step in this development process: moving beyond marker identification to the initial validation of a highly specific multi-locus diagnostic assay designed for clinical application.

Therefore, in this exploratory translational study, we evaluated the diagnostic accuracy and translational potential of a newly developed six-marker ctDNA methylation panel using quantitative PCR. The selected markers—Septin9, IKZF1, BCAT1, Septin9-2, BCAN, and VAV3—were chosen through a rigorous, data-driven process: methylation profiling of 595 cancer-related genomic targets in primary CRC, advanced adenoma, and normal tissues identified the top differentially methylated regions, from which the six most discriminatory and complementary loci were retained through iterative validation [17]. We specifically compared the performance of this assay with conventional CEA testing and, importantly, investigated the multi-locus nature of the ctDNA methylation burden as a potential biomarker of tumor extent and staging. The data from this prospective, single-center validation provide essential evidence for this novel assay’s progression toward a large-scale, pivotal clinical trial for CRC screening and surveillance.

## 2. Results

### 2.1. Baseline Characteristics

A total of 114 individuals were initially enrolled in the study. Of these, 22 samples were excluded due to quality control failures, including sample degradation and hemolysis. As a result, 92 participants were included in the final analysis: 57 healthy controls and 35 patients with CRC. Baseline characteristics of the study population are summarized in Table 1. The cohort included 62.0% males, with a mean age of 61.0 ± 7.4 years. Patients with CRC were significantly older than controls (64.8 ± 7.1 vs. 58.6 ± 6.5 years, *p* < 0.001). No significant differences were observed in sex distribution, body mass index (BMI), or smoking history across groups. Serum CEA levels were significantly higher in the CRC group (median 2.4 [IQR, 1.1–5.0] ng/mL) than in controls (1.6 [1.2–2.1] ng/mL, *p* = 0.046), and elevated CEA (>4.7 ng/mL) was more frequent among patients with CRC (25.7% vs. 1.8%, *p* = 0.001).

### 2.2. Diagnostic Performance of the ctDNA Methylation Assay

Among the 35 patients with CRC, 26 (74.3%) tested positive, whereas 7 (12.3%) of the 57 controls exhibited ctDNA positivity (Table 2), yielding a sensitivity of 74.3% and specificity of 87.7% using the platform-defined threshold of ≥1 positive marker.

**Figure 1:**
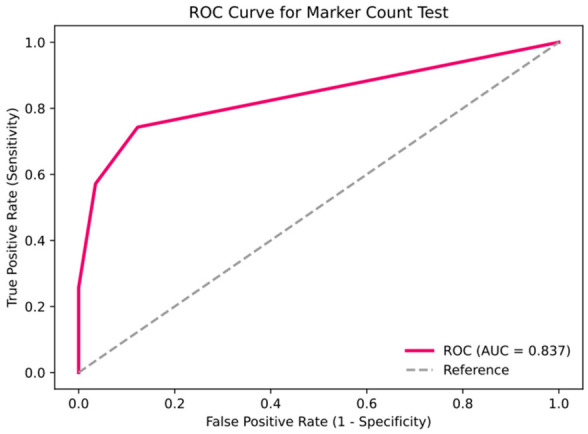
Receiver operating characteristic (ROC) curve for the marker-count diagnostic test. The solid magenta line depicts the ROC curve based on the number of positive markers (0–6) with an area under the curve (AUC) of 0.837. The dashed gray line represents the reference line (AUC = 0.5).

The receiver operating characteristic (ROC) curve analysis based on the number of methylation-positive markers (range, 0–6) demonstrated an AUC of 0.837, indicating good discriminatory capacity (Figure 1). The Youden’s index was maximized at a marker count of ≥1, reaching 0.620 (Supplementary Table S1), thereby supporting the predefined platform threshold. At a cut-off of three positive markers, the assay achieved 100% specificity. In comparison, serum CEA demonstrated lower sensitivity (25.7%) despite high specificity (98.2%).

Among the 57 controls, 24 (42.1%) had adenomatous polyps detected on screening colonoscopy, whereas 33 (57.9%) had no neoplastic lesions. When adenomas were considered separately within the control group, assay sensitivity was 20.8%, and specificity was 93.9%. Sensitivity of ctDNA detection increased progressively with pathological stage: 36.4% in stage I, 87.5% in stage II, 92.9% in stage III, and 100% in stage IV; these figures were derived by cross-referencing ctDNA test result with pathological stage as presented in Table 3 (Stage I: 4/11 = 36.4%; Stage II: 7/8 = 87.5%; Stage III: 13/14 = 92.9%; Stage IV: 2/2 = 100%).

### 2.3. Subgroup Analysis of Clinicopathologic Characteristics in Patient and Control Cohorts

Among the 35 patients with CRC, most had stage III disease (40.0%) with moderately differentiated tumors (62.9%) (Table 3). Patients who tested ctDNA-positive had significantly larger tumors (5.7 ± 2.0 cm vs. 2.2 ± 1.3 cm, *p* < 0.001) and were more likely to present with advanced T stage (*p* = 0.006), N stage (*p* = 0.03), and overall pathological stage (*p* = 0.007). In contrast, no significant differences were observed in BMI, sex distribution, or tumor location between ctDNA-positive and -negative patients. Other clinicopathologic factors, including elevated CEA, lymphovascular invasion (LVI), large vessel invasion (LaVI), perineural invasion (PNI), poor differentiation, and microsatellite instability (MSI) status, showed no statistically significant differences between ctDNA-positive and -negative patients, although higher-risk features were more frequent in the ctDNA-positive group.

In controls, no clinical differences were observed according to test positivity (Supplementary Table S2). In the false-positive group, 5 of 7 participants (71.4%) had adenomas detected during screening colonoscopy, compared with 19 (38.0%) in the test-negative group (*p* = 0.12).

### 2.4. Multi-Locus Methylation Patterns and Their Clinicopathologic Associations

**Figure 2:**
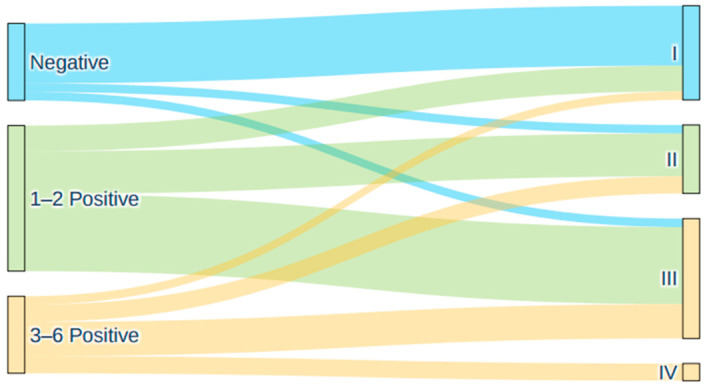
Sankey diagram illustrating the distribution of colorectal cancer stage according to the number of positive ctDNA methylation markers. Methylation categories are defined as follows: Negative = 0 of 6 markers positive; 1–2 Positive = 1 or 2 of 6 markers positive; 3–6 Positive = 3 or more of 6 markers positive. Flow width is proportional to patient count.

We further analyzed the distribution and clinicopathologic significance of individual methylation markers. The number of methylation-positive markers increased progressively with tumor stage (Table 4, Figure 2). Patients with stage I disease had a median of 0.0 [0.0–1.0] positive markers, compared with 5.5 [5.0–6.0] in stage IV (*p* = 0.003). The proportion of patients with ≥3 positive markers also increased across stages—from 9.1% in stage I, to 25.0% in stage II, 28.6% in stage III, and 100.0% in stage IV (*p* = 0.01). The proportion of Septin9.2 also significantly increased with stage increase (*p* = 0.009). In controls, no clinical differences were observed according to test positivity (Supplementary Table S2). In the false-positive group, 5 of 7 participants (71.4%) had adenomas detected during screening colonoscopy, compared with 19 (38.0%) in the test-negative group (*p* = 0.12).

**Figure 3:**
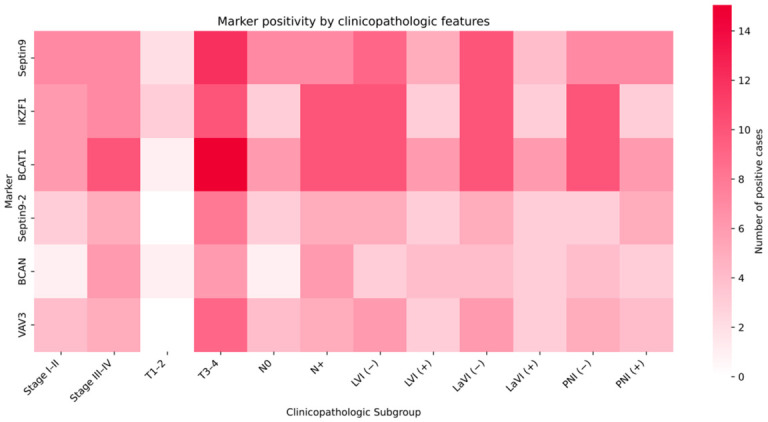
Heatmap of ctDNA methylation marker positivity across clinicopathologic subgroups. T = T stage; N = nodal positive status; LVI = lymphovascular invasion; LaVI = large vessel invasion; PNI = perineural invasion; ctDNA = circulating tumor DNA.

The distribution of individual marker expression by clinicopathologic features is shown in the heatmap (Figure 3) and Supplementary Table S3. BCAT1, Septin9-2, and VAV3 were significantly associated with advanced T stage (*p* = 0.01, 0.04, and 0.03, respectively). BCAN expression was significantly associated with nodal positivity (*p* = 0.03). In addition, BCAT1 was inversely associated with LaVI (*p* = 0.03); and Septin9-2 with PNI (*p* = 0.03).

## 3. Discussion

This prospective study evaluated the diagnostic performance of a six-marker ctDNA methylation assay for CRC detection. The assay demonstrated favorable diagnostic accuracy, with a sensitivity of 74.3%, specificity of 87.7%, and an AUC of 0.837. In comparison, traditional serum CEA showed markedly lower sensitivity (25.7%) despite excellent specificity (98.2%). Thus, the ctDNA assay provided a more robust and balanced diagnostic tool, with additional multi-locus benefits predicting tumor characteristics.

These findings suggest that this assay may complement or, in some cases, substitute for conventional diagnostic tools such as colonoscopy and stool-based tests. Colonoscopy remains the gold standard for CRC diagnosis and screening; however, its invasiveness, bowel preparation requirements, and associated patient discomfort limit compliance [20]. To address operator variability and training-related challenges, artificial intelligence assisted colonoscopy has been introduced, showing promise for improving adenoma detection. However, its application remains constrained by regional and economic disparities, and large-scale validation is still ongoing [21]. Stool-based tests, including FIT, FOBT, and fecal DNA testing, offer noninvasive alternatives but are hindered by modest sensitivity for early-stage disease, high false-positive rates, and low participation rates. In a study by Levi et al., compliance rates for FIT and FOBT were only 25.9% and 28.8%, respectively [22]. In Korea, although the national screening program includes FOBT, its use is limited in routine practice due to the widespread availability of colonoscopy. In our cohort, FOBT data were too scarce for meaningful analysis and were therefore excluded. By contrast, blood-based assays have demonstrated substantially higher patient acceptance [23] while also offering competitive diagnostic performance. Cai et al. reported a sensitivity of 86% and specificity of 92% for the same assay used in this study, compared with a sensitivity of 59.7% for FIT [17].

We acknowledge the reduced sensitivity observed for Stage I CRC (36.4%), which is a critical area for population screening. Cai et al. reported 85.7% sensitivity in stage I CRC [17]. This discrepancy with previous reports may stem from intrinsic limitations, such as the extremely low fractional abundance of ctDNA in early-stage disease, challenging even highly sensitive methylation assays [11]. To improve early-stage performance and maximize translational impact, future strategies must pivot toward a multi-modal approach. Integrating this methylation panel with other biomarkers (e.g., mutational profiling) or stool-based screening, particularly for high-risk populations, represents a promising pathway to enhance sensitivity while leveraging the stability and feasibility of methylation markers for routine use [8,24]. Overall, methylation-based biomarkers remain attractive due to their biological stability, feasibility for repeated testing, and potential for integration into multi-modal screening strategies [25].

A key novel translational finding of our study is the detailed evaluation of the multi-locus methylation burden as an indicator of tumor extent, moving beyond simple qualitative detection. We demonstrated a clear, intuitive relationship between the number of positive markers (categorized 0, 1–2, 3–6) and advancing tumor stage, providing a quantifiable severity score that directly correlates with clinicopathologic progression. While previous studies focused on single-marker positivity or quantitative levels of individual markers [26,27,28,29], our marker-count approach provides a readily interpretable, risk-stratification tool that is highly conducive to clinical decision-making. This finding underscores the potential for this multi-locus assay to be utilized not only for primary diagnosis but also for prognostic stratification or treatment intensity guidance.

Beyond overall methylation burden, the distinct associations of individual markers with specific pathological features (e.g., BCAN with nodal metastasis; BCAT1 and Septin9-2 with vascular invasion/PNI) are highly informative. These findings are consistent with prior studies. Symonds et al. demonstrated that BCAT1 and IKZF1 positivity increased with advancing T, N, and M stage, tumor size, and lymphatic invasion, whereas Uen et al. reported that VAV3 overexpression in CRC tissue was associated with higher rates of nodal and distant metastasis, although that study was not blood-based [30]. This suggests that the individual markers in our panel may capture discrete biological pathways in CRC progression. This marker-specific risk stratification potential is a powerful element for future personalized oncology applications, potentially guiding therapeutic selection in a translational setting.

A potential confounding factor warrants explicit consideration: the CRC group was significantly older than the control group (64.8 ± 7.1 vs. 58.6 ± 6.5 years; *p* < 0.001). Age-related epigenetic drift is well-documented and could theoretically inflate assay positivity in older individuals irrespective of cancer status [31]. To evaluate this, we performed a multivariate logistic regression adjusting for age, sex, and BMI. CRC status remained a strong independent predictor of ctDNA positivity (adjusted OR 16.81, 95% CI 5.24–53.96; *p* < 0.001), whereas age was not independently associated with assay positivity after adjustment for disease status (adjusted OR 1.05, 95% CI 0.97–1.13; *p* = 0.26), indicating that age-related methylation drift does not meaningfully drive false positivity in this assay (Supplementary Table S4).

We acknowledge the limitations inherent to this pilot exploratory translational study. The single-center design and modest sample size, particularly the underrepresentation of Stage IV disease, limit the generalizability of the findings and statistical power. However, the study’s primary translational intent was to evaluate the feasibility of this novel multi-locus assay and characterize its diagnostic features in a real-world Korean population before committing to a costly, large-scale randomized trial. This successful initial characterization provides the essential proof-of-concept required for future definitive validation. Future efforts should focus on multi-institutional, prospective longitudinal studies with larger cohorts to validate the prognostic utility and to test its performance in the minimal residual disease (MRD) setting, which is a high-impact translational application. Furthermore, integrated analysis combining this methylation testing with stool-based screening and other molecular features (e.g., mutational profiling) is warranted to maximize the diagnostic yield.

## 4. Materials and Methods

### 4.1. Study Groups and Test Procedure

This prospective case–control study was conducted at Asan Medical Center, a tertiary referral hospital in Seoul, Republic of Korea. Individuals aged 50–79 years were enrolled between 1 July 2024, and 1 January 2025. The study population included histologically confirmed patients with CRC who underwent radical or palliative surgical resection, as well as healthy controls who underwent colonoscopy for routine health screening. Exclusion criteria for both patients with CRC and controls included a prior history of malignancy, blood transfusion within the preceding two weeks, and pregnancy. Additional exclusions for the CRC group were prior neoadjuvant chemoradiation, previous endoscopic resection, or the presence of a secondary malignancy in the colon.

Peripheral blood (approximately 10 mL) was collected from all participants. For patients with CRC, samples were obtained preoperatively on the day of surgery. For controls, blood samples were drawn during outpatient visits prior to colonoscopy. All samples were transferred to the laboratory for processing, and those failing quality control, such as those affected by hemolysis, were excluded from the final analysis. ctDNA methylation analysis was performed using the ColonCatch^®^ platform (Clinomics Inc., Ulsan, Republic of Korea). Test positivity was defined as the presence of one or more methylation-positive markers. Laboratory personnel performing the ctDNA methylation assays received de-identified samples without access to participants’ clinical information. This study was approved by the Institutional Review Board of Asan Medical Center, University of Ulsan College of Medicine (IRB No. 2024-0412, 22 March 2024) and conducted in accordance with the Declaration of Helsinki. Written informed consent was obtained from all participants prior to enrollment. This study adhered to the Standards for Reporting Diagnostic Accuracy Studies guidelines by the EQUATOR network [32].

### 4.2. Data Collection

Demographic and clinical data were obtained through participants’ interviews and review of electronic medical records. Baseline variables included age, sex, smoking history, BMI, and serum CEA levels. CEA elevation was defined as >4.7 ng/mL, corresponding to the institution’s laboratory upper limit cutoff. For patients with CRC, additional data included tumor size, location, pathological TNM stage, LVI, LaVI, PNI, tumor budding, histologic differentiation, and MSI status, based on final pathology reports after resection. Pathologists who reviewed the surgical specimens and endoscopists who performed colonoscopy were unaware of the ctDNA test results.

### 4.3. Endpoints and Definitions

The primary endpoint was the diagnostic performance of the ctDNA methylation assay, specifically sensitivity and specificity for CRC detection. Secondary endpoints included the relationship between ctDNA test positivity (defined as ≥1 positive marker) and clinicopathologic features, as well as the distribution and count of methylation-positive markers according to pathological stage. Associations between individual markers and pathologic variables were also analyzed.

### 4.4. Statistical Analysis

Continuous variables were compared using Student’s *t*-test or the Mann–Whitney U test and categorical variables using the Chi-square test or Fisher’s exact test, as appropriate. Diagnostic accuracy was assessed using ROC curve analysis, with AUC values reported. To assess the potential confounding effect of age on ctDNA positivity, multivariate logistic regression was performed with ctDNA test result as the binary outcome and CRC status, age, sex, and BMI as covariates. All statistical analyses were performed using R software (version 4.3.4; R Foundation for Statistical Computing, Vienna, Austria). Selected visualizations, including Sankey diagrams and ROC curves, were generated using Plotly v.6.8 (Plotly Technologies Inc., Montréal, QC, Canada) in a Python 3.10 environment via Google Colaboratory (Google LLC., Mountain View, CA, USA). A two-sided *p*-value < 0.05 was considered statistically significant.

## 5. Conclusions

In conclusion, this exploratory translational study provides the initial validation for a novel six-marker ctDNA methylation assay, confirming its potential as a robust molecular diagnostic tool for colorectal cancer. The assay demonstrated reliable diagnostic accuracy (AUC 0.837) and significantly outperformed traditional serum CEA.

Crucially, we established that the multi-locus methylation burden, defined by the number of positive markers, provides a quantifiable measure of tumor extent that correlates significantly with advancing tumor stage and invasive features. This finding supports the translational utility of the assay beyond primary diagnosis, suggesting strong potential for prognostic risk stratification and postoperative surveillance. While sensitivity for early-stage CRC remains a technical challenge, these data provide the essential proof-of-concept for the assay’s technical robustness and clinical relevance. Future multi-institutional, large-scale prospective studies are warranted to validate its efficacy when integrated into multi-modal screening strategies, paving the way for its ultimate clinical adoption.

## Figures and Tables

**Table 1:** Baseline characteristics and ctDNA test positivity of the study population.

	Total (n = 92)	Study Cohort		
		Control (n = 57)	CRC (n = 35)	*p*
**Sex**				0.33
Male	57 (62.0)	38 (66.7)	19 (54.3)	
Female	35 (38.0)	19 (33.3)	16 (45.7)	
**Age, years**	61.0 ± 7.4	58.6 ± 6.5	64.8 ± 7.1	<0.001
**BMI (kg/m^2^) [IQR]**	23.8 [21.7; 25.5]	24.2 [22.7; 25.9]	22.9 [21.3; 24.9]	0.10
**Smoking history**				0.62
Never	44 (47.8)	25 (43.9)	19 (54.3)	
Current	18 (19.6)	12 (21.1)	6 (17.1)	
Past	30 (32.6)	20 (35.1)	10 (28.6)	
**CEA (ng/mL) [IQR]**	1.7 [1.1; 2.5]	1.6 [1.2; 2.1]	2.4 [1.1; 5.0]	0.046
Elevated CEA (>4.7 ng/mL)	10 (10.9)	1 (1.8)	9 (25.7)	0.001

Values are presented as a number (%), mean ± SD, or median [IQR]. CRC = colorectal cancer; BMI = body mass index; CEA = carcinoembryonic antigen; IQR = interquartile range; SD = standard deviation.

**Table 2:** ctDNA test positivity rates among control and colorectal cancer subgroups.

	Control Non-Neoplastic (n = 33)	Control Adenoma (n = 24)	Colorectal Adenocarcinoma (n = 35)	*p*
**ctDNA test**				<0.001
Negative	31 (93.9)	19 (79.2)	9 (25.7)	
Positive	2 (6.1)	5 (20.8)	26 (74.3)	

Values are presented as a number (%). ctDNA positivity was defined as ≥1 of 6 methylation markers positive (Septin9, IKZF1, BCAT1, Septin9-2, BCAN, VAV3).

**Table 3:** Clinicopathologic features according to ctDNA test result among patients with colorectal cancer.

	Total (n = 35)	ctDNA Test		*p*
		Negative (n = 9)	Positive (n = 26)	
**Sex**				0.46
Male	19 (54.3)	6 (66.7)	13 (50.0)	
Female	16 (45.7)	3 (33.3)	13 (50.0)	
**Age, years**	64.8 ± 7.1	61.4 ± 9.1	66.0 ± 6.1	0.10
**BMI (kg/m^2^)**	23.2 ± 3.2	23.4 ± 3.3	23.2 ± 3.2	0.86
**CEA (ng/mL) [IQR]**	2.4 [1.1; 5.0]	1.1 [1.0; 3.3]	2.8 [1.4; 7.6]	0.14
Elevated CEA (>4.7 ng/mL)	9 (25.7)	1 (11.1)	8 (30.8)	0.39
**Tumor size (cm)**	4.8 ± 2.4	2.2 ± 1.3	5.7 ± 2.0	<0.001
**Tumor location**				>0.99
Right	10 (28.6)	2 (22.2)	8 (30.8)	
Left	25 (71.4)	7 (77.8)	18 (69.2)	
**Pathological T stage**				0.006
T1	7 (20.0)	5 (55.6)	2 (7.7)	
T2	4 (11.4)	2 (22.2)	2 (7.7)	
T3	20 (57.1)	2 (22.2)	18 (69.2)	
T4	4 (11.4)	0 (0.0)	4 (15.4)	
**Pathological N stage**				0.03
N0	19 (54.3)	8 (88.9)	11 (42.3)	
N1	10 (28.6)	0 (0.0)	10 (38.5)	
N2	6 (17.1)	1 (11.1)	5 (19.2)	
**Pathological stage**				0.007
I	11 (31.4)	7 (77.8)	4 (15.4)	
II	8 (22.9)	1 (11.1)	7 (26.9)	
III	14 (40.0)	1 (11.1)	13 (50.0)	
IV	2 (5.7)	0 (0.0)	2 (7.7)	
**Lymphovascular** **invasion**	8 (22.9)	0 (0.0)	8 (30.8)	0.08
**Large vessel invasion**	7 (20.0)	0 (0.0)	7 (26.9)	0.15
**Perineural invasion**	10 (28.6)	1 (11.1)	9 (34.6)	0.24
**Tumor budding**				0.59
High	6 (17.1)	1 (11.1)	5 (19.2)	
Intermediate	4 (11.4)	0 (0.0)	4 (15.4)	
Low	25 (71.4)	8 (88.9)	17 (65.4)	
**Differentiation**				0.11
Well-differentiated	10 (28.6)	5 (55.6)	5 (19.2)	
Moderately differentiated	22 (62.9)	4 (44.4)	18 (69.2)	
Mucinous	3 (8.6)	0 (0.0)	3 (11.5)	
**MSI status**				>0.99
MSI-High	1 (2.9)	0 (0.0)	1 (3.8)	
MSI-Low	5 (14.3)	1 (11.1)	4 (15.4)	
MSS	29 (82.9)	8 (88.9)	21 (80.8)	

Values are presented as a number (%) or as a mean ± standard deviation unless otherwise indicated. BMI = body mass index; CEA = carcinoembryonic antigen; IQR = interquartile range; MSI = microsatellite instability. ctDNA positivity was defined as ≥1 of 6 methylation markers positive (Septin9, IKZF1, BCAT1, Septin9-2, BCAN, VAV3).

**Table 4:** Distribution of ctDNA methylation positivity and marker expression by pathological stage.

	Total (n = 35)	Pathologic Stage				*p*
		I (n = 11)	II (n = 8)	III (n = 14)	IV (n = 2)	
**Number of positive markers**						
0 (negative)	9 (25.7)	7 (63.6)	1 (12.5)	1 (7.7)	0 (0.0)	0.01
1–2	17 (48.6)	3 (27.3)	5 (62.5)	9 (64.3)	0 (0.0)	
3–6	9 (25.7)	1 (9.1)	2 (25.0)	4 (28.6)	2 (100.0)	
**Septin9**	14 (40.0)	2 (18.2)	5 (62.5)	5 (35.7)	2 (100.0)	0.07
**IKZF1**	13 (37.1)	2 (18.2)	4 (50.0)	5 (35.7)	2 (100.0)	0.13
**BCAT1**	16 (45.7)	2 (18.2)	4 (50.0)	8 (57.1)	2 (100.0)	0.09
**Septin9.2**	8 (22.9)	0 (0.0)	3 (37.5)	3 (21.4)	2 (100.0)	0.009
**BCAN**	7 (20.0)	0 (0.0)	1 (12.5)	5 (35.7)	1 (50.0)	0.07
**VAV3**	9 (25.7)	1 (9.1)	3 (37.5)	3 (21.4)	2 (100.0)	0.06

Values are presented as a number (%).

## Data Availability

The original contributions presented in this study are included in the article/Supplementary Material. Further inquiries can be directed to the corresponding author.

## Supplementary Materials

The following supporting information can be downloaded at: https://www.mdpi.com/article/10.3390/ijms27135738/s1.

## Abbreviations

The following abbreviations are used in this manuscript:

AUC	Area under the curve
CRC	Colorectal cancer
FOBT	Fecal occult blood testing
FIT	Fecal immunochemical test
CEA	Carcinoembryonic antigen
ctDNA	Circulating tumor DNA
LVI	Lymphovascular invasion
LaVI	Large vessel invasion
PNI	Perineural invasion
MSI	Microsatellite instability
ROC	Receiver operating characteristic
