# Supplementary material for: Translational Validation of a Novel Multi-Locus ctDNA Methylation Assay for Early Detection and Stratification of Colorectal Cancer: An Exploratory Prospective, Case-Control Study
Source: Int J Mol Sci. 2026 Jun 25;27(13):5738. doi: 10.3390/ijms27135738 (PMC13362356; doi:10.3390/ijms27135738)
Supplement: Supplementary file 1 [file ijms-27-05738-s001.zip › ijms-4371635-supplementary.pdf]

**Supplementary Table S1. Diagnostic performance metrics by number of methylation-positive markers**

| Cutoff (≥ marker count) | Sensitivity (%) | Specificity (%) | Youden's Index |
|---|---|---|---|
| **≥ 0** | 100.0 | 0.0 | 0.0 |
| **≥ 1** | 74.3 | 87.7 | 0.62 |
| **≥ 2** | 57.1 | 96.5 | 0.536 |
| **≥ 3** | 25.7 | 100.0 | 0.257 |
| **≥ 4** | 17.1 | 100.0 | 0.171 |
| **≥ 5** | 8.6 | 100.0 | 0.086 |
| **≥ 6** | 5.7 | 100.0 | 0.057 |

**Supplementary Table S2. Clinical features among controls according to ctDNA test result**

| | Total (n=57) | ctDNA test Negative (n=50) | ctDNA test Positive (n=7) | *p* |
|---|---|---|---|---|
| **Sex** | | | | >0.99 |
| Male | 38 (66.7) | 33 (66.0) | 5 (71.4) | |
| Female | 38 (66.7) | 33 (66.0) | 5 (71.4) | |
| **Age, years** | 64.8 ± 7.1 | 58.7 ± 6.6 | 58.1 ± 6.6 | 0.83 |
| **BMI (kg/m$^2$)** | 23.2 ± 3.2 | 24.6 ± 5.1 | 25.5 ± 3.5 | 0.64 |
| **CEA (ng/mL) [IQR]** | 1.6 [1.2; 2.1] | 1.6 [1.2; 2.0] | 1.6 [1.4; 2.5] | 0.49 |
| Elevated CEA (> 4.7 ng/mL) | 1 (1.8) | 1 (2.0) | 0 (0.0) | >0.99 |
| **Adenoma detection** | 24 (42.1) | 19 (38.0) | 5 (71.4) | 0.12 |
| **Number of positive markers** | | | | |
| 1 | NA | NA | 5 (71.4) | |
| 2 | NA | NA | 2 (28.6) | |
| **Smoking status** | | | | > 0.99 |
| Never | 25 (43.9) | 22 (44.0) | 3 (42.9) | |
| Current | 12 (21.1) | 11 (22.0) | 1 (14.3) | |
| Past | 20 (35.1) | 17 (34.0) | 3 (42.9) | |

Values are presented as a number (%) or as a mean ± standard deviation unless otherwise indicated. BMI = Body Mass Index; CEA = carcinoembryonic antigen; NA = not applicable; INR = interquartile range.

**Supplementary Table S3. Association between individual methylation markers and clinicopathologic features**

| Marker | Septin9 (n = 14) | *p* | IKZF1 (n = 13) | *p* | BCAT1 (n = 16) | *p* | Septin9-2 (n = 8) | *p* | BCAN (n = 7) | *p* | VAV3 (n = 9) | *p* |
|---|---|---|---|---|---|---|---|---|---|---|---|---|
| **T stage** | | 0.14 | | 0.48 | | 0.01 | | 0.04 | | 0.39 | | 0.03 |
| T1–2 | 2 (14.3) | | 3 (23.1) | | 1 (6.2) | | 0 (0.0) | | 1 (14.3) | | 0 (0.0) | |
| T3–4 | 12 (85.7) | | 10 (76.9) | | 15 (93.8) | | 8 (100.0) | | 6 (85.7) | | 9 (100.0) | |
| **N stage** | | 0.95 | | 0.70 | | 0.14 | | 0.42 | | 0.03 | | 0.70 |
| N0 | 7 (50.0) | | 3 (23.1) | | 6 (37.5) | | 3 (37.5) | | 1 (14.3) | | 4 (44.4) | |
| N+ | 7 (50.0) | | 10 (76.9) | | 10 (62.5) | | 5 (62.5) | | 6 (85.7) | | 5 (55.6) | |
| **Pathological stage** | | 0.95 | | 0.70 | | 0.14 | | 0.42 | | 0.03 | | 0.70 |
| I–II | 7 (50.0) | | 6 (46.2) | | 6 (37.5) | | 3 (37.5) | | 1 (14.3) | | 4 (44.4) | |
| III–IV | 7 (50.0) | | 7 (53.8) | | 10 (62.5) | | 5 (62.5) | | 6 (85.7) | | 5 (55.6) | |
| **LVI** | | 0.22 | | > 0.99 | | 0.11 | | 0.35 | | 0.03 | | 0.40 |
| No | 9 (64.3) | | 10 (76.9) | | 10 (62.5) | | 5 (62.5) | | 3 (42.9) | | 6 (66.7) | |
| Yes | 5 (35.7) | | 3 (23.1) | | 6 (37.5) | | 3 (37.5) | | 4 (57.1) | | 3 (33.3) | |
| **LaVI** | | 0.40 | | > 0.99 | | 0.03 | | 0.31 | | 0.13 | | 0.34 |
| No | 10 (71.4) | | 10 (76.9) | | 10 (62.5) | | 5 (62.5) | | 4 (57.1) | | 6 (66.7) | |
| Yes | 4 (28.6) | | 3 (23.1) | | 6 (37.5) | | 3 (37.5) | | 3 (42.9) | | 3 (33.3) | |
| **PNI** | | 0.05 | | 0.71 | | 0.45 | | 0.03 | | 0.38 | | 0.39 |
| No | 7 (50.0) | | 10 (76.9) | | 10 (62.5) | | 3 (37.5) | | 4 (57.1) | | 5 (55.6) | |
| Yes | 7 (50.0) | 3 (37.5) | 3 (23.1) | | 6 (37.5) | | 5 (62.5) | | 3 (42.9) | | 4 (44.4) | |

Values are presented as a number (%) or as a mean ± standard deviation unless otherwise indicated. T = T stage; N = nodal positive status; LVI = lymphovascular invasion; LaVI = Large vessel invasion; PNI = perineural invasion.

**Supplementary Table S4.** Logistic regression analysis for predictors of ctDNA positivity

| Variable | Univariate | | Multivariate | |
|---|---|---|---|---|
| | **OR (95% CI)** | **p** | **OR (95% CI)** | **p** |
| CRC | 20.63 (6.90–61.72) | <0.001 | 16.81 (5.24–53.96) | <0.001 |
| Age (per year) | 1.11 (1.04–1.18) | 0.002 | 1.05 (0.97–1.13) | 0.260 |
| Sex (male) | 0.62 (0.26–1.47) | 0.275 | 0.72 (0.22–2.34) | 0.580 |
| BMI (kg/m$^2$) | 0.96 (0.86–1.07) | 0.457 | 1.03 (0.91–1.17) | 0.611 |

Outcome: ctDNA positivity (positive vs. negative). Multivariate model adjusted for all listed variables simultaneously. OR = odds
